# The Relationship Between Thyroid Function and Metabolic Syndrome and Its Components: A Cross-Sectional Study in a Chinese Population

**DOI:** 10.3389/fendo.2021.661160

**Published:** 2021-03-31

**Authors:** Jiaji He, Yaxin Lai, Jing Yang, Yongli Yao, Yongze Li, Weiping Teng, Zhongyan Shan

**Affiliations:** ^1^ Department of Endocrinology and Metabolism, Institute of Endocrinology, The First Affiliated Hospital of China Medical University, Shenyang, China; ^2^ Department of Endocrinology, The First Hospital of Shanxi Medical University, Taiyuan, China; ^3^ Department of Endocrinology, Qinghai Provincial People’s Hospital, Xining, China

**Keywords:** thyroid function, hyperthyroidism, hypothyroidism, metabolic syndrome, hypertriglyceridemia, hyperglycaemia

## Abstract

**Objective:**

The present study examined the relationship between thyroid function status and the prevalence of metabolic syndrome in a Chinese population.

**Methods:**

Cross-sectional data were obtained from the Thyroid Disease, Iodine Nutrition and Diabetes Epidemiology (TIDE) Survey. A total of 62,408 subjects aged ≥18 years were enrolled. Differences in metabolic indicators and the prevalence of metabolic syndrome according to sex and thyroid function status were compared. Logistic regression was used to analyze the influence of thyroid function on metabolic syndrome and its components.

**Results:**

The prevalence of metabolic syndrome was generally higher in men than women. Overt hyperthyroidism and subclinical hypothyroidism had a significant effect on metabolism in men. Body mass index (BMI), waist circumference, and triglycerides (TGs) were significantly lower in men in the overt hyperthyroidism group, and BMI, waist circumference, systolic blood pressure (SBP) and TGs were higher in men in the subclinical hypothyroidism group than men in the normal group. Overt and subclinical hypothyroidism had significant impacts on metabolic components in women. BMI, waist circumference, TGs, SBP and DBP in the subclinical and overt hypothyroidism groups were significantly higher than the euthyroid group in women. The relative risk of abdominal obesity and hypertriglyceridemia was increased in women with hypothyroidism. Thyroid dysfunction had different effects on metabolic syndrome and its components before and after menopause.

**Conclusion:**

Thyroid function had important effects on the prevalence of metabolic syndrome. Women with hypothyroidism, especially post-menopausal women, had a higher risk of metabolic syndrome than men.

## Introduction

Metabolic syndrome comprises a group of interrelated metabolic abnormalities that are characterized by central obesity, high triglycerides (TGs), low high-density lipoprotein cholesterol (HDL-C), hypertension and hyperglycaemia. Patients with metabolic syndrome have an increased risk of cardiovascular disease, type 2 diabetes, and all-cause mortality. After adjusting for potential risk factors and each component of metabolic syndrome as a continuous variable, metabolic syndrome was associated with an increased 10-year risk of coronary heart disease (1). It is estimated that the population attributable rates of metabolic syndrome are approximately 6%-7% for all-cause mortality, 12%-17% for cardiovascular disease, and 30%-52% for diabetes (2).

With the development of the social economy in recent decades, the incidence rates of nutritional metabolic diseases, such as obesity, hypertension and diabetes, have significantly increased (3). A survey from China showed that the prevalence of metabolic syndrome among Chinese adults increased in recent years, and it has become a major public health problem (4–7). The incidence of metabolic syndrome in urban areas is higher than rural areas, and the overall prevalence tends to increase with age. Sex heterogeneity exists in the relationship between risk factors and the prevalence of metabolic syndrome (7, 8). Economic development, urbanization, improvements in living standards, changes in lifestyle, dietary modifications and a reduction in physical activities all play key roles in this process (4).

The thyroid plays an important role in metabolic regulation. Thyroid hormones have multiple effects on glucose and lipid metabolism, blood pressure regulation, and energy consumption. Recent studies found that patients with hypothyroidism and subclinical hypothyroidism had an increased risk of metabolic syndrome (9, 10). Previous studies showed that subjects with thyroid stimulating hormone (TSH) levels at the upper limit of the normal range (2.5-4.5 mU/L) had increased rates of obesity, increased TG levels, and an increased possibility of metabolic syndrome (11). Healthy young women with TSH levels > 2.5 mU/L should be evaluated for the presence of metabolic syndrome, even if TSH levels are within the normal range (12). Other reports did not show a significant association between high TSH levels and metabolic syndrome (13, 14). Obesity also affects thyroid function. A meta-analysis of 22 studies showed a significant association between obesity and an increased risk of hypothyroidism (RR = 1.86), and obese people had an increased risk of overt hypothyroidism (RR=3.21) and subclinical hypothyroidism (RR=1.70) (15). This relationship requires further investigation in a representative large-sample population.

There is increasing evidence that thyroid dysfunction affects lipid and glucose metabolism, blood pressure, and body weight, which are associated with various metabolic parameters and may lead to the development or aggravation of components of metabolic syndrome (16). The present cross-sectional study investigated the association between thyroid dysfunction and metabolic syndrome in a Chinese population.

## Materials and Methods

### Study Design

The data were obtained from the Thyroid Disease, Iodine Nutrition and Diabetes Epidemiology (TIDE) study, which included urban and rural areas, and were obtained *via* four-stage random sampling (17). The following inclusion criteria for adult respondents were used: aged 18 years or older, living in a target community for at least 5 years, no exposure to iodine or contrast agent in the previous three months, and not pregnant. The Ethics Committee of China Medical University approved the research plan. After a detailed explanation of the protocol, all respondents signed informed consent forms.

The questionnaire collected data on demographics, personal and family histories of thyroid disease, smoking status, family income, education level and household salt consumption. Fasting blood and urine were collected from each subject, and blood samples were collected from subjects without diagnosed diabetes after the 2-h oral glucose tolerance test (OGTT). The collected serum and urine samples were stored at -20°C. After investigation and specimen collection, all samples were transported to the central laboratory and adhered to cold chain requirements for the unified testing of thyroid indexes and urinary iodine concentration (UIC). Metabolic indexes were detected immediately on site.

### Laboratory Testing and Clinical Diagnosis

Fasting blood glucose (FBG), 2-hour blood glucose (OGTT 2-hPG), serum TG, total cholesterol (TC), low-density lipoprotein cholesterol (LDL-C) and HDL-C levels were measured using an automatic biochemical analyser (BS180 analyzer, Mindray, Shenzhen, China). HbA1c in venous blood samples was measured using high-performance liquid chromatography (HPLC) (BioRad VARIANT II haemoglobin analyser). Thyroid stimulating hormone (TSH), thyroid peroxidase antibody (TPOAb) and thyroglobulin antibody (TgAb) were measured using electrochemical luminescence immunoassays (Cobasc601 analyser, Roche Diagnostic, Switzerland). When the TSH level exceeded the upper limit of the reference range (0.27-4.20 mIU/L), free thyroxine (FT4) was measured. FT4 and free triiodothyronine (FT3) were measured when TSH levels were lower than the lower limit of the reference range. UIC was measured using inductively coupled plasma mass spectrometry (ICP-MS) (Agilent 7700x, Agilent Technologies, USA).

According to the International Diabetes Federation (IDF) consensus worldwide definition of the metabolic syndrome promulgated in 2005 in combination with the actual situation of the Chinese population, metabolic syndrome was diagnosed when 3 or more of the following criteria were present: ① abdominal obesity (male waist ≥90 cm, female waist ≥85 cm); ② hypertriglyceridemia (TG≥1.7 mmol/L (150 mg/dL)); ③ low HDL-C (< 1.0 mmol/L in males, < 1.3 mmol/L in females); ④ hypertension (the presence of any of the following 3 conditions was defined as abnormal blood pressure: (a) antihypertensive treatment, (b) systolic blood pressure (SBP) 130 mmHg or higher, or (c) diastolic blood pressure (DBP) ≥85 mmHg); and ⑤ hyperglycaemia (the presence of any of the following 3 conditions was defined as abnormal blood glucose: (a) a self-reported diabetes history or hypoglycaemic treatment, (b) fasting plasma glucose (FPG) ≥5.6 mmol/L (100 mg/dL), (c) OGTT 2-hPG ≥7.8 mmol/L (140 mg/dL), or (d) HbA1c ≥5.7%) (18, 19).

### Statistical Analysis

All statistical analyses were performed using SPSS 22.0 (IBM, USA). Corresponding participants was randomly selected from a normal thyroid function population as the control group to eliminate the influence of quantity differences. Normally distributed data are expressed as means ± standard deviations. Two independent samples t-tests were used to compare differences in metabolic indicators. The chi-squared and Fisher’s exact test were used to compare differences in the prevalence of metabolic syndrome. Logistic regression was performed to calculate adjusted odds ratios (ORs) and 95% confidence intervals (CIs) to analyze the effects of thyroid function on metabolic syndrome and its components and the effects before and after menopause. Two types of risk factor adjustment models were constructed. A P-value less than 0.05 was considered statistically significant.

## Results

### General Characteristics of Participants

**Figure 1:**
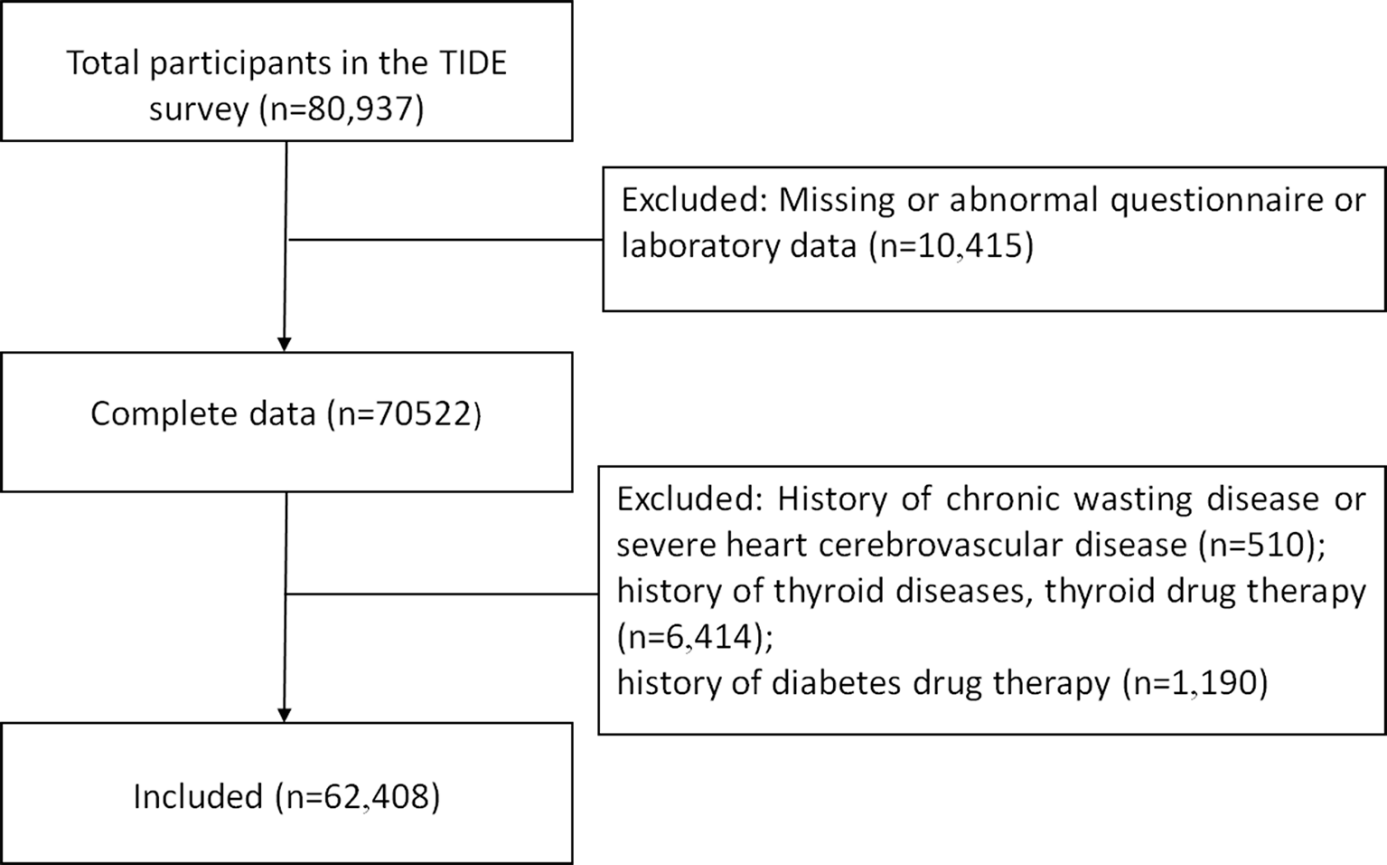
Screening flow chart.

A total of 80,937 participants were enrolled after excluding participants who met the exclusion criteria, and 62,408 participants were ultimately included in the analyses. The flow chart of participant inclusion is shown in **Figure 1**. A total of 52,755 participants (84.5%) had normal thyroid function, 342 patients had overt hyperthyroidism, 199 patients had subclinical hyperthyroidism, 581 patients had overt hypothyroidism, and 8,531 patients had subclinical hypothyroidism. In particular, there are 7 patients with central hypothyroidism among the patients with low TSH. To eliminate the influence of differences in group sizes, 9,950 participants were randomly selected from the normal group as the euthyroid control group. The general characteristics of participants with different thyroid function statuses are shown in **Table 1**.

**Table 1:** General characteristics of participants with different thyroid functional statuses.

	EUT	SHyper	Ohyper	Shypo	Ohypo
***Demographic characteristics***					
Number	9950	199	342	8531	581
Sex, Male/Female (%/%)	5268/4682 (52.9/47.16)	74/125 (37.2/62.8)	142/200 (41.5/58.5)	3395/5136 (39.8/60.2)	149/432 (25.6/74.4)
Age	41.87 ± 15.17	46.01 ± 15.98	41.53 ± 13.72	45.29 ± 16.15	50.98 ± 15.12
Ethnicity (%)					
Han	9031 (90.8)	163 (81.9)	308 (90.1)	7577 (88.8)	459 (79.0)
Others	919 (9.2)	36 (18.1)	34 (9.9)	954 (11.2)	122 (21.0)
Education n (%)					
Senior high school or lower level	6488 (65.6)	147 (73.9)	238 (69.6)	6128 (71.8)	489 (84.2)
College or higher level	3426 (34.4)	52 (26.1)	104 (30.4)	2403 (28.2)	92 (15.8)
Location n(%)					
Urban	5134 (51.6)	82 (41.2)	163 (47.7)	4196 (49.2)	240 (41.3)
Rural	4816 (48.4)	117 (58.8)	179 (52.3)	4335 (50.8)	341 (58.7)
Profession n(%)					
Peasant	2469 (24.8)	75 (37.7)	111 (32.5)	2824 (33.1)	257 (44.2)
Office staff	2260 (22.7)	40 (20.1)	60 (17.5)	1671 (19.6)	81 (13.9)
Worker or others	5221 (52.5)	84 (42.2)	171 (50.0)	4036 (47.3)	243 (41.9)
Income n(%)					
<50000 yuan	6587 (66.2)	157 (78.9)	248 (72.5)	6066 (71.1)	463 (79.7)
>=50000 yuan	3363 (33.8)	42 (21.1)	94 (27.5)	2465 (28.9)	118 (20.3)
Iodized-salt intake n(%)					
YES	9509 (95.6)	193 (97.0)	332 (97.1)	8268 (96.9)	558 (96.0)
NO	441 (4.4)	6 (3.0)	10 (2.9)	263 (3.1)	23 (4.0)
Smoker n(%)					
YES	2898 (29.1)	49 (24.6)	83 (24.3)	1567 (18.4)	86 (14.8)
NO	7052 (70.9)	150 (75.4)	259 (75.7)	6964 (81.6)	495 (85.2)
Family history of thyroid diseases n (%)					
YES	508 (5.1)	10 (5.0)	21 (6.1)	330 (3.9)	24 (4.1)
NO	9442 (94.9)	189 (95.0)	321 (93.9)	8201 (96.1)	557 (95.9)
Family history of diabetes n(%)					
YES	1494 (15.0)	23 (11.6)	39 (11.4)	1100 (12.9)	58 (10.0)
NO	8456 (85.0)	176 (88.4)	303 (88.6)	7431 (87.1)	523 (90.0)
**Metabolic Index**					
BMI (kg/m^2^)	23.92 ± 3.71	23.73 ± 3.61	23.39 ± 4.01	24.03 ± 3.79	24.66 ± 3.49
Waist (cm)	83.02 ± 10.97	82.47 ± 11.12	81.14 ± 10.57	82.78 ± 10.86	84.15 ± 10.37
SBP (mmHg)	124.91 ± 18.81	128.31 ± 20.63	126.02 ± 18.37	126.44 ± 19.39	128.95 ± 19.61
DBP (mmHg)	77.98 ± 12.45	78.40 ± 13.07	77.18 ± 11.48	78.83 ± 12.48	79.51 ± 12.52
FBG (mmol/L)	5.22 ± 1.04	5.19 ± 1.02	5.25 ± 0.78	5.17 ± 0.98	5.23 ± 0.88
OGTT 2-hPG (mmol/L)	6.4137 ± 2.69	6.29 ± 2.25	6.49 ± 2.21	6.33 ± 2.57	6.42 ± 2.48
HbA1c (%)	5.42 ± 1.26	5.37 ± 1.01	5.37 ± 0.88	5.44 ± 1.31	5.52 ± 0.81
TGs (mmol/L)	1.55 ± 1.36	1.37 ± 0.82	1.31 ± 0.79	1.61 ± 1.37	1.67 ± 1.34
HDL-C (mmol/L)	1.48 ± 0.54	1.70 ± 0.78	1.49 ± 0.59	1.47 ± 0.52	1.58 ± 0.74
**Thyroid-related indexes**					
TSH (mIU/mL)	2.21 ± 0.88	0.11 ± 0.09	0.05 ± 0.06	6.14 ± 2.78	21.04 ± 26.76
FT4(pmol/L)	–	17.42 ± 2.38	36.63 ± 28.56	15.96 ± 2.15	10.11 ± 2.28
FT3(pmol/L)	–	5.44 ± 0.80	12.95 ± 9.14	–	–
TPOAb (mIU/mL)	22.13 ± 7.95	81.93 ± 70.14	144.54 ± 74.53	56.06 ± 33.80	182.20 ± 29.33
TgAb (mIU/mL)	46.96 ± 19.84	182.62 ± 70.65	325.23 ± 75.11	122.41 ± 47.43	414.52 ± 96.92
UIC (ug/L)	250.52 ± 76.43	247.82 ± 68.43	325.89 ± 60.21	307.34 ± 48.50	240.26 ± 47.64

EUT, normal thyroid function, Shyper, subclinical hyperthyroidism, Ohyper, overt hyperthyroidism, Shypo, subclinical hypothyroidism, Ohypo, overt hypothyroidism, BMI, body mass index, SBP, systolic blood pressure, DBP, diastolic blood pressure, FBG, fasting blood glucose, OGTT, oral glucose tolerance test, HbA1c, glycosylated haemoglobin, TG, triglyceride, HDL-C, high-density lipoprotein cholesterol, TSH, thyroid stimulating hormone, TPOAb, thyroid peroxidase antibody, TgAb, thyroglobulin antibody, UIC, urinary iodine concentration.

### Metabolic Differences in Different Thyroid Function Statuses in Men and Women

The metabolic indicators are related to sex. Therefore, we compared differences in metabolic indicators in different thyroid functional statuses in men and women.

As shown in **Table 2**, SBP and HDL-C were increased in men in the subclinical hyperthyroidism group compared to men the euthyroid group, and the TG level was reduced. BMI, waist circumference, and TG levels were significantly reduced in the overt hyperthyroidism group. BMI, waist circumference, SBP and TG level were increased in the subclinical hypothyroidism group, and SBP and HDL-C were increased in the overt hypothyroidism group.

**Table 2:** Metabolic differences in different thyroid functional states by sex.

	EUT	SHyper	OHyper	SHypo	OHypo
Men					
N	5268	74	142	3395	149
age	41.58 ± 15.36	48.89 ± 16.00^**^	42.77 ± 14.21	45.08 ± 17.04	53.48 ± 16.36^**^
BMI (kg/m^2^)	24.45 ± 3.66	24.46 ± 3.77	23.68 ± 3.76^*^	24.60 ± 3.74^*^	24.73 ± 3.48
Waist (cm)	86.38 ± 10.43	86.88 ± 11.91	84.50 ± 10.83^*^	86.67 ± 10.68^*^	86.35 ± 10.36
SBP (mmHg)	128.62 ± 17.53	136.22 ± 22.57^**^	130.37 ± 16.50	129.74 ± 18.21^*^	132.99 ± 20.56^**^
DBP (mmHg)	80.25 ± 12.81	82.34 ± 13.34	79.64 ± 11.80	81.06 ± 12.98	82.13 ± 13.76
FBG (mmol/L)	5.27 ± 1.15	5.16 ± 0.83	5.25 ± 0.80	5.22 ± 1.09	5.39 ± 0.93
OGTT 2-hPG (mmol/L)	6.43 ± 2.94	6.33 ± 2.29	6.26 ± 2.29	6.32 ± 2.73	6.42 ± 2.39
HbA1c (%)	5.45 ± 1.55	5.51 ± 0.95	5.37 ± 0.89	5.48 ± 1.79	5.47 ± 0.83
TGs (mmol/L)	1.76 ± 1.55	1.42 ± 0.73^**^	1.43 ± 0.81^**^	1.81 ± 1.58^**^	1.73 ± 1.37
HDL-C (mmol/L)	1.37 ± 0.48	1.70 ± 0.89^**^	1.37 ± 0.54	1.34 ± 0.47	1.55 ± 0.71^**^
Women					
N	4682	125	200	5136	432
Age	42.20 ± 14.94	44.30 ± 15.78	41.36 ± 13.39	45.43 ± 15.53^**^	50.11 ± 14.58^**^
BMI (kg/m^2^)	23.33 ± 3.67	23.30 ± 3.46	23.18 ± 4.18	23.66 ± 3.77^**^	24.63 ± 3.50^**^
Waist (cm)	79.25 ± 10.33	79.86 ± 9.77	78.75 ± 9.72	81.53 ± 10.38^**^	83.39 ± 10.28^**^
SBP (mmHg)	120.73 ± 19.32	123.62 ± 17.90	122.93 ± 19.03	124.26 ± 19.83^**^	127.56 ± 19.10^**^
DBP (mmHg)	75.42 ± 11.51	76.02 ± 12.35	75.43 ± 10.74	77.36 ± 11.91^**^	78.60 ± 11.95^**^
FBG (mmol/L)	5.15 ± 0.91	5.21 ± 1.12	5.25 ± 0.78	5.13 ± 0.91	5.17 ± 0.86
OGTT 2-hPG (mmol/L)	6.38 ± 2.39	6.27 ± 2.23	6.65 ± 2.14	6.34 ± 2.46	6.43 ± 2.51
HbA1c (%)	5.39 ± 0.82	5.29 ± 1.03	5.36 ± 0.88	5.42 ± 0.87	5.53 ± 0.81^**^
TGs (mmol/L)	1.32 ± 1.05	1.34 ± 0.87	1.23 ± 0.76	1.48 ± 1.19^**^	1.64 ± 1.32^**^
HDL-C (mmol/L)	1.61 ± 0.57	1.70 ± 0.70	1.56 ± 0.61	1.56 ± 0.53^**^	1.59 ± 0.76

Compared to the euthyroid group.

*P ＜ 0.05, **P ＜ 0.01.

BMI, waist circumference, SBP, DBP, and TG levels in the subclinical and overt hypothyroidism groups were significantly increased in the subclinical hypothyroidism group compared to women in the euthyroid group, and HDL-C was significantly decreased. HbA1c was significantly increased in the overt hypothyroidism group.

### Prevalence of Metabolic Syndrome and Each of Its Component in Men and Women by Thyroid Function Status

**Figure 2:**
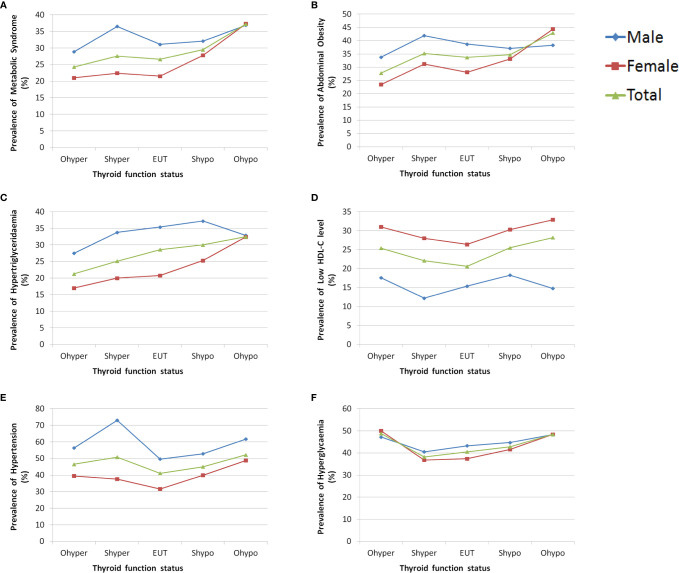
The prevalence of metabolic syndrome and each of its component in different thyroid function status groups by sex. **(A)** Prevalence of metabolic syndrome grouped by sex. **(B)** Prevalence of abdominal obesity grouped by sex. **(C)** Prevalence of hypertriglyceridemia grouped by sex. **(D)** Prevalence of low HDL-C grouped by sex. **(E)** Prevalence of hypertension grouped by sex. **(F)** Prevalence of hyperglycaemia grouped by sex.

The prevalence of metabolic syndrome was significantly higher in men than women (30.8% vs. 23.2%, P < 0.01). The prevalence of metabolic syndrome and each of its component in the different thyroid function status groups are shown in **Figure 2**. Among the different thyroid function groups, the prevalence of hypertension in men was consistently higher than women, the prevalence of low HDL-C was consistently significantly higher in women than men, and the prevalence of hyperglycaemia was similar between men and women. The prevalence of metabolic syndrome, abdominal obesity and hypertriglyceridemia in men with overt hypothyroidism, subclinical hyperthyroidism, euthyroid, and subclinical hypothyroidism were higher than the corresponding groups of women. However, differences were not observed in the overt hypothyroidism group.

### Analysis of the Risk of Metabolic Syndrome Associated With Thyroid Function Using Logistic Regression

The associations of thyroid function with metabolic syndrome and its components were analyzed using binary logistic regression according to sex and thyroid function status (**Table 3**). Model 1 was constructed using univariate analysis, and Model 2 was adjusted for the effects of age, ethnicity, education, occupation, annual income, smoking history, and other metabolic factors.

**Table 3:** Risk of metabolic syndrome associated with thyroid function in men and women.

	SHyper	OHyper	SHypo	OHypo
**Men**				
**Abdominal obesity**	1.168 (0.735,1.854)	0.826 (0.583,1.170)	0.948 (0.880,1.020)	1.003 (0.720,1.397)
	1.276 (0.769,2.115)	0.949 (0.656,1.375)	0.889 (0.819,1.014)	1.011 (0.701,1.458)
**Hypertriglyceridemia**	0.947 (0.585,1.535)	0.702 (0.485,1.016)	1.115 (1.035,1.200)^**^	0.910 (0.646,1.281)
	0.949 (0.575,1.566)	0.716 (0.490,1.048)	1.205 (1.115,1.302)^**^	0.975 (0.682,1.396)
**Low HDL-C**	0.772 (0.384,1.551)	1.193 (0.744,1.840)	1.283 (1.169,1.409)^**^	0.966 (0.614,1.521)
	0.812 (0.403,1.638)	1.229 (0.795,1.900)	1.373 (1.249,1.509)^**^	1.103 (0.698,1.742)
**Hypertension**	2.683 (1.605,4.483)^**^	1.281 (0.919,1.786)	1.121 (1.044,1.204)^*^	1.604 (1.152,2.233)^*^
	2.477 (1.426,4.303)^**^	1.610 (1.129,2.296)^**^	1.019 (0.943,1.102)	1.109 (0.780,1.578)
**Hyperglycaemia**	0.889 (0.559,1.415)	1.166 (0.838,1.622)	1.060 (0.987,1.139)	1.221 (0.884,1.684)
	0.677 (0.410,1.120)	1.492 (1.047,2.126)^*^	0.902 (0.833,1.005)	0.740 (0.523,1.047)
**Metabolic syndrome**	1.291 (0.804,2.073)	0.911 (0.633,1.311)	1.068 (0.989,1.153)	1.316 (0.942,1.836)
	1.190 (0.732,1.935)	0.951 (0.655,1.380)	1.028 (0.950,1.112)	1.105 (0.784,1.556)
**Women**				
**Abdominal obesity**	1.084 (0.742,1.584)	0.733 (0.528,1.017)	1.221 (1.145,1.302)^**^	1.931 (1.595,2.338)^**^
	1.094 (0.718,1.668)	0.849 (0.599,1.203)	1.080 (1.007,1.157)^*^	1.453 (1.183,1.783)^**^
**Hypertriglyceridemia**	0.912 (0.588,1.415)	0.746 (0.516,1.080)	1.291 (1.204,1.384)^**^	1.766 (1.441,2.164)^**^
	0.898 (0.568,1.419)	0.748 (0.511,1.095)	1.183 (1.099,1.274)^**^	1.452 (1.172,1.799)^**^
**Low HDL-C**	1.047 (0.708,1.548)	1.211 (0.896,1.635)	1.211 (1.134,1.293)^**^	1.323 (1.081,1.620)^**^
	1.082 (0.729,1.606)	1.191 (0.879,1.613)	1.189 (1.113,1.271)^**^	1.262 (1.028,1.549)^*^
**Hypertension**	1.204 (0.838,1.730)	1.306 (0.982,1.735)	1.403 (1.319,1.492)^**^	1.925 (1.592,2.328)^**^
	1.172 (0.779,1.764)	1.614 (1.182,2.205)^**^	1.190 (1.110,1.277)^**^	1.310 (1.059,1.620)^*^
**Hyperglycaemia**	0.926 (0.683,1.332)	1.595 (1.208,2.107)^**^	1.162 (1.093,1.235)^*^	1.499 (1.240,1.813)^**^
	0.893 (0.596,1.336)	2.181 (1.616,2.943)^**^	0.938 (0.876,1.004)	0.980 (0.794,1.208)
**Metabolic syndrome**	0.960 (0.630,1.462)	0.883 (0.628,1.242)	1.353 (1.265,1.448)^**^	1.998 (1.641,2.432)^**^
	0.868 (0.545,1.382)	0.992 (0.689,1.428)	1.133 (1.052,1.221)^**^	1.409 (1.137,1.747)^**^

*P < 0.05, **P < 0.01.

Model 1, Single-factor analysis.

Model 2, Adjusted for confounding factors (age, ethnicity, education level, occupation, annual income, smoking history, other metabolic factors, etc.).

Subclinical hyperthyroidism in men was a risk factor for hypertension. Overt hyperthyroidism was a risk factor for hypertension and hyperglycaemia. Subclinical hypothyroidism was a risk factor for hypertriglyceridemia and low HDL-C. Overt hypothyroidism had no effect on metabolic syndrome or its components.

Overt hyperthyroidism in women was a risk factor for hypertension and hyperglycaemia. Subclinical hypothyroidism was a risk factor for abdominal obesity, hypertriglyceridemia, low HDL-C, hypertension and metabolic syndrome. Overt hypothyroidism was a risk factor for abdominal obesity, hypertriglyceridemia, low HDL-C, hypertension and metabolic syndrome. In general, subclinical hypothyroidism and overt hypothyroidism were risk factors for metabolic syndrome. However, subclinical hyperthyroidism had no effect on metabolic syndrome or its components.

TSH levels were divided into quartiles in the euthyroid control group, and the association between TSH levels and components of the metabolic syndrome were analyzed (**Table 4**). The risk of metabolic syndrome in men increased with TSH levels at the lower limit of the normal range (0.27-1.25 mIU/L). The risk of abdominal obesity in women increased significantly with TSH levels at the upper limit of the normal range (3.21-4.20mIU/L).

**Table 4:** Risk of metabolic syndrome associated with TSH levels in the euthyroid group.

TSH level (mIU/L)	0.27-1.25	1.25-2.23	2.23-3.21	3.21-4.20
**Men**				
**Abdominal obesity**	1.216 (0.916,1.615)	1.054 (0.921,1.205)	0.965 (0.818,1.139)	1.010 (0. 803,1.270)
	1.196 (0.876,1.634)	1.036 (0.893,1.202)	1.010 (0.843,1.211)	1.037 (0.805,1.337)
**Hypertriglyceridemia**	1.143 (0.851,1.535)	1.056 (0.921,1.212)	0.906 (0.766,1.073)	1.065 (0.845,1.342)
	1.011 (0.738,1.385)	1.074 (0.926,1.247)	0.916 (0.763,1.099)	1.071 (0.830,1.382)
**Low HDL-C**	1.517 (0.976,2.358)	1.062 (0.885,1.275)	0.890 (0.711,1.115)	0.948 (0.704,1.277)
	1.287 (0.816,2.032)	1.065 (0.883,1.285)	0.908 (0.720,1.145)	1.919 (0.676,1.249)
**Hypertension**	0.993 (0.755,1.305)	1.033 (0.906,1.177)	1.108 (0.944,1.301)	1.058 (0.846,1.323)
	1.207 (0.895,1.627)	1.068 (0.927,1.229)	1.179 (0.991,1.402)	0.958 (0.753,1.218)
**Hyperglycaemia**	0.814 (0.617,1.068)	1.005 (0.881,1.147)	0.980 (0.832,1.153)	1.124 (0.897,1.408)
	0.902 (0.669,1.217)	1.048 (0.906,1.213)	1.025 (0.857,1.226)	0.992 (0.775,1.270)
**Metabolic syndrome**	1.285 (0.948,1.740)	1.003 (0.870,1.156)	0.934 (0.784,1.112)	0.969 (0.764,1.231)
	1.398 (1.025,1.907)^*^	1.047 (0.905,1.212)	0.966 (0.807,1.157)	0.898 (0.701,1.149)
**Women**				
**Abdominal obesity**	0.654 (0.476,0.897)^**^	0.999 (0.850,1.175)	1.121 (0.940,1.336)	1.485 (1.189,1.854)^**^
	0.960 (0.668,1.379)	1.044 (0.870,1.252)	1.080 (0.889,1.312)	1.532 (1.193,1.967)^**^
**Hypertriglyceridemia**	0.840 (0.588,1.201)	1.089 (0.950,1.310)	1.069 (0.881,1.298)	1.190 (0.933,1.517)
	1.236 (0.5835,1.830)	1.113 (0.914,1.357)	1.047 (0.851,1.289)	1.118 (0.862,1.451)
**Low HDL-C**	1.113 (0.785,1.580)	1.058 (0.894,1.251)	1.152 (0.966,1.375)	1.235 (0.986,1.546)
	1.246 (0.866,1.793)	1.048 (0.882,1.245)	1.153 (0.962,1.381)	1.153 (0.914,1.455)
**Hypertension**	0.592 (0.435,0.804)^**^	1.088 (0.929,1.273)	1.144 (0.965,1.357)	0.990 (0.798,1.229)
	0.930 (0.651,1.330)	1.167 (0.973,1.399)	1.093 (0.901,1.327)	0.873 (0.682,1.117)
**Hyperglycaemia**	0.619 (0.459,0.833)^**^	0.836 (0.719,0.972)^*^	1.037 (0.880,1.221)	1.065 (0.864,1.314)
	0.826 (0.591,1.154)	0.816 (0.689,1.011)	0.951 (0.790,1.143)	0.985 (0.777,1.248)
**Metabolic syndrome**	0.631 (0.448,0.888)^**^	0.945 (0.791,1.132)	1.179 (0.973,1.429)	1.258 (0.994,1.593)
	1.054 (0.718,1.547)	1.004 (0.824,1.224)	1.137 (0.919,1.221)	1.238 (0.958,1.600)

*P < 0.05, **P < 0.01.

Model 1, Single-factor analysis.

Model 2, Adjusted for confounding factors (age, ethnicity, education level, occupation, annual income, smoking history, other metabolic factors, etc.).

### The Influence of Female Menopause on the Relationship Between Thyroid Function and Metabolic Syndrome

The female population was further divided into pre- and post-menopausal groups, and binary logistic regression was used to investigate the effects of changes in thyroid function on the risk of metabolic syndrome before and after menopause (**Table 5**). After adjusting for age, ethnicity, education, occupation, annual income, smoking history, and other metabolic factors, overt hyperthyroidism was a risk factor for hypertension and hyperglycaemia in women before menopause. However, the effect of overt hyperthyroidism disappeared after menopause. Subclinical hypothyroidism increased the risk of abdominal obesity, hypertension, hypertriglyceridemia, low HDL-C and metabolic syndrome before menopause, but these effects were not observed after menopause. Subclinical hypothyroidism was associated with hypertension before and after menopause. Overt hypothyroidism was significantly associated with abdominal obesity, hypertriglyceridemia and metabolic syndrome before menopause, and these effects persisted after menopause.

**Table 5:** Risk of metabolic syndrome associated with thyroid function in women before and after menopause.

	SHyper	OHyper	SHypo	OHypo
**Abdominal obesity**				
** before**	1.154 (0.676,1.971)	0.692 (0.447,1.072)	1.152 (1.047,1.268)^**^	1.191 (1.067,1.313)^**^
** after**	0.923 (0.501,1.700)	0.752 (0.420,1.346)	0.946 (0.854,1.047)	1.711 (1.284,2.281)^**^
**Hypertriglyceridemia**				
** before**	0.746 (0.383,1.453)	0.732 (0.456,1.175)	1.315 (1.188,1.455)^**^	1.375 (1.031,1.835)^*^
** after**	1.125 (0.592,2.139)	0.714 (0.70,1.379)	1.059 (0.952,1.179)	1.526 (1.113,2.093)^**^
**Low HDL-C**				
** before**	1.247 (0.773,2.010)	1.036 (0.721,1.490)	1.282 (1.179,1.395)^**^	1.328 (1.001,1.765)^*^
** after**	0.818 (0.401,1.667)	1.656 (0.929,2.952)	1.086 (0.973,1.211)	1.227 (0.912,1.650)
**Hypertension**				
** before**	1.248 (0.739,2.106)	1.650 (1.138,2.392)^**^	1.213 (1.105,1.332)^**^	1.343 (0.997,1.809)
** after**	1.146 (0.604,2.175)	1.673 (0.925,3.027)	1.143 (1.028,1.272)^*^	1.305 (0.964,1.767)
**Hyperglycaemia**				
** before**	0.817 (0.477,1.399)	2.318 (1.643,3.270)^**^	0.935 (0.855,1.022)	1.020 (0.761,1.367)
** after**	1.048 (0.554,1.984)	1.809 (0.989,3.310)	0.929 (0.836,1.033)	0.924 (0.687,1.243)
**Metabolic syndrome**				
** before**	0.969 (0.500,1.877)	0.927 (0.571,1.505)	1.308 (1.176,1.455)^**^	1.451 (1.051,2.002)^*^
** after**	0.837 (0.446,1.571)	1.113 (0.627,1.977)	0.974 (0.880,1.079)	1.445 (1.021,1.872)^*^

*P < 0.05, **P < 0.01.

## Discussion

The prevalence of cardiovascular events and stroke and the risk of death in Chinese patients with metabolic syndrome are 2 ~ 3 times higher than Chinese people without metabolic syndrome. Non-diabetic patients with metabolic syndrome have a five-fold increased risk of developing type 2 diabetes compared to nondiabetic patients without metabolic syndrome. Early intervention and diagnosis of metabolic syndrome are key in the prevention of diabetes and cardiovascular and cerebrovascular diseases. Accumulated evidence suggests that thyroid dysfunction affects lipid and glucose metabolism, blood pressure, and body weight, which are associated with various metabolic parameters and may lead to the development or deterioration of components of metabolic syndrome (16).

The present cross-sectional study based on TIDE data found that thyroid function status affected metabolic syndrome and its components differently in the general population. The prevalence of metabolic syndrome in men was generally higher than women, and the prevalence of metabolic syndrome in women with overt hypothyroidism was highest. Overt hyperthyroidism had a more obvious effect on men’s metabolism than women’s metabolism. Women’s metabolic status was more sensitive to overt and subclinical hypothyroidism than men’s metabolic status. Further studies found that the effect of hypothyroidism on the prevalence of metabolic syndrome in women was primarily due to changes in lipid metabolism and increased risks of abdominal obesity and hypertriglyceridemia. Overall, women with hypothyroidism have a higher risk of metabolic syndrome than men. Our study further found different effects of thyroid dysfunction on metabolic syndrome and its components before and after menopause. The effects of overt hyperthyroidism, subclinical hypothyroidism and overt hypothyroidism on metabolic syndrome and its components primarily occurred in women before menopause. Postmenopausal women with subclinical hypothyroidism had increased risks of hypertension and abdominal obesity, and women with overt hypothyroidism had increased risks of hypertriglyceridemia and metabolic syndrome.

Most previous studies suggested that increased serum TSH levels were associated with metabolic syndrome. Lee Yeo Kyung et al. found that serum TSH concentrations within the normal reference range were significantly positively correlated with the prevalence of metabolic syndrome in Korea (20). Bensenor Isabela M et al. examined 10,935 participants from Brazil and found that high TSH was closely related to metabolic syndrome (21). However, Huang CY et al. found that serum TSH levels were not correlated with metabolic syndrome, a relatively high serum T3 concentration had a strong correlation with metabolic syndrome, and a relatively low serum T4 concentration had no obvious correlation with metabolic syndrome (22). Differences in these results may be explained by the fact that most of these studies evaluated the effects of TSH level on metabolic syndrome rather than thyroid dysfunction as a whole. One section of the study analyzed only components of metabolic syndrome associated with serum TSH levels, and another section analyzed TSH, FT3 and FT4 as variables. The relationship between TSH, FT3, and FT4 and metabolic parameters is complex and may be limited by age, sex, nationality and many other factors. Because the distribution of these factors in different populations varies greatly, the conclusions cannot generalized to the general population.

Metabolic syndrome is subject to sex heterogeneity, and the disease characteristics in men and women are different. Our results showed that abnormal lipid metabolism may be the key reason for the difference in the risk of metabolic syndrome between the two sexes. Especially in postmenopausal women, overt and subclinical hypothyroidism primarily affected the risk of metabolic syndrome by increasing the risks of abdominal obesity (OR=1.711) and hypertriglyceridemia (OR=1.526). The secretion of sex hormones changes significantly in perimenopausal women. Oestrogen secretion is decreased due to altered ovarian function, and the secretion of androgens from the adrenal cortex is only mildly affected. Sex hormones influence lipid accumulation patterns and differ between men and women. Premenopausal women are prone to peripheral obesity, accompanied by hypodermic and adipose accumulation, and men and postmenopausal women easily accumulate abdominal fat, resulting in central obesity. The risks of abdominal obesity, cardiovascular disease-related mortality, and the development of type 2 diabetes increase (23). There is a sex difference in the relationship between thyroid dysfunction and lipid level (24, 25). After menopause, women have increased levels of follicle-stimulating hormone (FSH). Serum FSH levels are positively correlated with serum TC levels, and the incidence of hypercholesterolemia in perimenopausal women is significantly higher than premenopausal women (26). Although subclinical hypothyroidism is associated with obesity and hypertriglyceridemia in women before menopause, the correlation disappeared in women after menopause, which suggests that the effects of gonadotropin on the components of metabolic syndrome in women may be stronger than TSH.

The mechanism of the association between thyroid dysfunction and metabolic syndrome is not fully understood. Insulin resistance due to thyroid dysfunction may be an important cause and basis of metabolic syndrome. Hyperthyroidism may result in insulin resistance because hyperthyroidism is associated with the catabolism of excessive thyroid hormones, which may affect components of metabolic syndrome, such as body weight and lipid levels, and hypothyroidism is associated with reduced insulin sensitivity. The explanation for this apparent contradiction may lie in the different effects of thyroid hormones on the liver and peripheral tissues (27). Thyroid hormones simultaneously act as insulin agonists (in the muscle) and antagonists (in the liver) in different organs, and excess thyroid hormone (or possibly deficiency) disrupts the balance and leads to hepatic insulin resistance primarily resulting in increased glucose output and glycogen decomposition and glucose intolerance. However, thyroid function is also associated with lipid metabolism. Patients with overt hypothyroidism and subclinical hypothyroidism are prone to disorders of blood lipid metabolism. Hypothyroidism causes an increase in weight. TSH is positively correlated with BMI and obesity. TSH and BMI increase simultaneously in obese patients (28), and the prevalence of hypertriglyceridemia and low HDL-C level also increases (10, 29). The effect on HDL-C may be due to changes in the activity of liver lipase and cholesterol ester transfer protein (30).

Our study has several advantages. First, the data in this study were obtained from the TIDE project database, which covers all provinces in mainland China and had a sufficient sample size and representativeness. This study analyzed the relationship between thyroid function and metabolic syndrome from the perspective of thyroid function status: normal thyroid function, overt hyperthyroidism, subclinical hyperthyroidism, subclinical hypothyroidism, and overt hypothyroidism.

There are many limitations in this study. First, serum TSH was measured in all participants, and FT4 and FT3 were measured in only some patients with abnormal thyroid function. Insulin and sex hormone levels were not measured. Therefore, it was not possible to individually analyze these influencing factors. Second, because this study was a cross-sectional study rather than a cohort study, the causal inference between thyroid function and metabolic syndrome cannot be confirmed, and further prospective studies should be performed to elucidate causality.

In conclusion, thyroid dysfunction was associated with metabolic syndrome, and the association differed by sex. Overt hypothyroidism and subclinical hypothyroidism were associated with an increased risk of metabolic syndrome, especially in postmenopausal women. The change may be due to the effect of TSH on blood lipids. To determine the significance of early detection of thyroid dysfunction, particularly in the subclinical form, and the long-term association with metabolic syndrome in different age, sex, and BMI groups, large-population follow-up cohort studies and studies with longer follow-up periods are needed. Reasonable, early interventions should be performed in women with hypothyroidism and menopausal women.

## Data Availability Statement

The datasets generated for this study are available on request to the corresponding authors.

## Ethics Statement

The studies involving human participants were reviewed and approved by Medical Ethics Committee of China Medical University. The patients/participants provided their written informed consent to participate in this study.

## Author Contributions

JH, YaL, JY, and YY contributed equally to this work. JH, YaL, and YoL performed the data analyses and drafted the manuscript. JY, YY, and the Thyroid Disorders, Iodine Status and Diabetes Epidemiological Survey Group participated in the epidemiological investigations. WT and ZS conceived and designed the study and interpreted the results. All authors contributed to the article and approved the submitted version.

## Funding

This work was supported by the Research Fund for Public Welfare from National Health and Family Planning Commission of China (Grant No. 201402005) and the Clinical Research Fund of Chinese Medical Association (Grant No. 15010010589).

## Conflict of Interest

The authors declare that the research was conducted in the absence of any commercial or financial relationships that could be construed as a potential conflict of interest.
